# Mapping the Genes for Susceptibility and Response to *Leishmania tropica* in Mouse

**DOI:** 10.1371/journal.pntd.0002282

**Published:** 2013-07-11

**Authors:** Yahya Sohrabi, Helena Havelková, Tetyana Kobets, Matyáš Šíma, Valeriya Volkova, Igor Grekov, Taťána Jarošíková, Iryna Kurey, Jarmila Vojtíšková, Milena Svobodová, Peter Demant, Marie Lipoldová

**Affiliations:** 1 Laboratory of Molecular and Cellular Immunology, Institute of Molecular Genetics, Academy of Sciences of the Czech Republic, Prague, Czech Republic; 2 Faculty of Biomedical Engineering, Czech Technical University in Prague, Kladno, Czech Republic; 3 Faculty of Science, Charles University, Prague, Czech Republic; 4 Roswell Park Cancer Institute, Buffalo, New York, United States of America; Yale School of Public Health, United States of America

## Abstract

**Background:**

*L. tropica* can cause both cutaneous and visceral leishmaniasis in humans. Although the *L. tropica*-induced cutaneous disease has been long known, its potential to visceralize in humans was recognized only recently. As nothing is known about the genetics of host responses to this infection and their clinical impact, we developed an informative animal model. We described previously that the recombinant congenic strain CcS-16 carrying 12.5% genes from the resistant parental strain STS/A and 87.5% genes from the susceptible strain BALB/c is more susceptible to *L. tropica* than BALB/c. We used these strains to map and functionally characterize the gene-loci regulating the immune responses and pathology.

**Methods:**

We analyzed genetics of response to *L. tropica* in infected F_2_ hybrids between BALB/c×CcS-16. CcS-16 strain carries STS-derived segments on nine chromosomes. We genotyped these segments in the F_2_ hybrid mice and tested their linkage with pathological changes and systemic immune responses.

**Principal Findings:**

We mapped 8 *Ltr* (*Leishmania tropica* response) loci. Four loci (*Ltr2*, *Ltr3*, *Ltr6* and *Ltr8*) exhibit independent responses to *L. tropica*, while *Ltr1*, *Ltr4*, *Ltr5* and *Ltr7* were detected only in gene-gene interactions with other *Ltr* loci. *Ltr3* exhibits the recently discovered phenomenon of transgenerational parental effect on parasite numbers in spleen. The most precise mapping (4.07 Mb) was achieved for *Ltr1* (chr.2), which controls parasite numbers in lymph nodes. Five *Ltr* loci co-localize with loci controlling susceptibility to *L. major*, three are likely *L. tropica* specific. Individual *Ltr* loci affect different subsets of responses, exhibit organ specific effects and a separate control of parasite load and organ pathology.

**Conclusion:**

We present the first identification of genetic loci controlling susceptibility to *L. tropica*. The different combinations of alleles controlling various symptoms of the disease likely co-determine different manifestations of disease induced by the same pathogen in individual mice.

## Author Summary

Leishmaniasis, a disease caused by *Leishmania* ssp. is among the most neglected infectious diseases. In humans, *L. tropica* causes cutaneous form of leishmaniasis, but can damage internal organs too. The reasons for this variability are not known, and its genetic basis was never investigated. Therefore, analysis of genes affecting host's responses to this infection can elucidate the characteristics of individual host-parasite interactions. Recombinant congenic strain CcS-16 carries 12.5% genes from the mouse strain STS/A on genetic background of the strain BALB/c, and it is more susceptible than BALB/c. In F_2_ hybrids between BALB/c and CcS-16 we detected and mapped eight gene-loci, *Ltr1-8* (*Leishmania tropica* response 1-8) that control various manifestations of disease: skin lesions, splenomegaly, hepatomegaly, parasite numbers in spleen, liver, and inguinal lymph nodes, and serum level of CCL3, CCL5, and CCL7 after *L. tropica* infection. These loci are functionally heterogeneous - each influences a different set of responses to the pathogen. Five loci co-localize with the previously described loci that control susceptibility to *L. major*, three are species-specific. *Ltr2* co-localizes not only with *Lmr14* (*Leishmania major* response 14), but also with *Ir2* influencing susceptibility to *L. donovani* and might therefore carry a common gene controlling susceptibility to leishmaniasis.

## Introduction

Leishmaniasis is endemic in 98 countries on 5 continents, causing 20,000 to 40,000 deaths per year [1]. In the past decade the number of endemic regions have expanded, prevalence has increased and the number of unrecorded cases must have been substantial, because notification has been compulsory in only 32 of the 98 countries where 350 million people are at risk [1], [2]. Infection represents an important global health problem, as no safe and effective vaccine currently exists against any form of human leishmaniasis, and the treatment is hampered by serious side effects [3].

The disease is caused by obligate intracellular vector-borne parasites of the genus *Leishmania*. In the vertebrate host organism, *Leishmania* parasites infect so-called professional phagocytes (neutrophils, monocytes and macrophages) [4], as well as dendritic cells [5], immature myeloid precursor cells, sialoadhesin-positive stromal macrophages of the bone marrow, hepatocytes and fibroblasts [6]. Leishmaniasis includes asymptomatic infection and three main clinical syndromes. In the dermis, parasites cause the cutaneous form of the disease, which can be localized or diffuse; in the mucosa, they cause mucocutaneous leishmaniasis, and the metastatic spread of infection to the spleen and liver leads to visceral leishmaniasis (also known as kala-azar or black fever). Parasites can also enter other organs, such as lymph nodes, bone marrow and lungs, and in rare cases, can even reach the brain [4]. One of the major factors determining the type of pathology is the species of *Leishmania* [7]. However, the transmitting vector, as well as genotype, nutritional status of the host, and environmental and social factors also have a large impact on the outcome of the disease [4], [7]. That is why even patients infected by the same species of *Leishmania* develop different symptoms [7] and may differ in response to therapy [3]. The basis of this heterogeneity is not well understood [8], but part of this variation is likely genetic [4].

The search for loci and genes controlling leishmaniasis included candidate-gene approach, genome-wide linkage and association mapping. Genotyping of candidate genes, which have been chosen on the basis of previous immunological studies (hypothesis-driven approach) detected influence of polymorphism in *HLA-Cw7, HLA-DQw3, HLA-DR, TNFA* (tumor necrosis factor alpha), *TNFB, IL4, IFNGR1* (interferon gamma receptor 1) [reviewed in [4]], *TGFB1* (transforming growth factor, beta 1) [9], *IL1* [10], *IL6* [11], *CCL2/MCP1* (chemokine (C-C motif) ligand 2) [12], *CXCR1* (chemokine (C-X-C motif) receptor 1) [13], *CXCR2* (chemokine (C-X-C motif) receptor 2) [14], *FCN2* (ficolin-2) [15] and *MBL2* (mannose-binding lectin (protein C) 2) [16] on response to different human leishmaniases.

Hypothesis-independent search for susceptibility genes included genome-wide linkage and association mapping. Bucheton and coworkers [17] performed a genome-wide linkage scan, identified a major susceptibility locus that controls the susceptibility to *L. donovani* on chromosome 22q12 [17] and found that polymorphism in *IL2RB* (interleukin 2 receptor, beta chain) in this chromosomal region is associated with susceptibility to visceral leishmaniasis [18]. Genome-wide search with the subsequent analysis of a putative susceptibility locus on chromosome 6q27 revealed that polymorphism in *DLL1* (delta-like 1 (Drosophila)), the ligand for NOTCH3 (Neurogenic locus notch homolog protein 3) [19] is associated with susceptibility to visceral leishmaniasis caused by *L. donovani* and *L. infantum chagasi*. Delta1-Notch3 interactions bias the functional differentiation of activated CD4^+^ T cells [20]. GWAS (genome-wide association study) established that common variants in the *HLA-DRB1-HLA-DQA1* HLA class II region contribute to susceptibility to *L. donovani* and *L. infantum chagasi* [21].

Genome-wide linkage in mouse revealed susceptibility genes *Nramp1* (Natural resistance-associated macrophage protein 1)/*Slc11a1* (solute carrier family 11 (proton-coupled divalent metal ion transporters), member 1) [22] and *Fli1* (Friend leukaemia virus integration 1) [23] and the role of these genes has been also established in humans [13], [24], [25]. *NRAMP1*, which controls susceptibility to *L. donovani* and *L. infantum* functions as a divalent metal pH-dependent efflux pump at the phagosomal membrane of macrophages and neutrophils [26]. It is also expressed in dendritic cells and influences major histocompatibility complex class II expression and antigen-presenting cell function [27]. Susceptible mouse allele carries a “null” mutation that abolishes gene function (it is a natural knockout) [28], whereas polymorphisms in the promoter, exon3 and the intron of human *SLC11A1* [24], are expected to have a smaller impact on gene function. The Friend leukaemia virus integration gene, linked with wound healing, influences cutaneous leishmaniasis caused by *L. major* in mouse [23] and by *L. braziliensis* in human [25]. It remains to be tested, whether natural polymorphisms detected in mouse genes *bg* (beige)/*Lyst* (lysosomal trafficking regulator) [29] and cationic amino acid transporter *Slc7a2* (solute carrier family 7 (cationic amino acid transporter, y+ system), member 2) [30] influencing response to *L. donovani* [31] and *L. major* [30], respectively, plays role also in humans. However, nothing is known about genes controlling *L. tropica*-induced disease in humans.

*L. tropica* causes cutaneous leishmaniasis in humans, but it can also visceralize. Although cutaneous disease due to *L. tropica* is known for a long time, its potential to visceralize in humans has been recognized only relatively recently [32]. Visceralized *L. tropica* was also identified as the cause of an initially not understood systemic illness in veterans returning from endemic areas in the Middle East [33]. This finding stimulated interest in less typical symptoms induced by this parasite. It was found that *L. tropica* caused visceral disease in Kenya [34], as well as classical visceral leishmaniasis (kala-azar) in India [35], [36] and in Iran [37], and disseminated cutaneous leishmaniasis accompanied with visceral leishmaniasis in Iran [38]. *L. tropica* was also implicated in development of mucosal leishmaniasis in Iran [39]. The reasons of this variability are not known.

A suitable animal model for study of this parasite would therefore contribute to genetic dissection of the functional and clinical manifestations of infection. Golden hamsters (*Mesocricetus auratus*) have been considered to be the best model host for *L. tropica* infection, but this host is not inbred and therefore not suitable for genetic dissection. Fortunately, several *L. tropica* strains from Afghanistan, India [40], and Turkey [41] have been reported to cause cutaneous disease in inbred BALB/c mice. Extension of analysis to the strains C57BL/6J, C57BL/10SgSnAi and gene-deficient mice on their backgrounds indicated role of IL-10 and TGFβ in regulation of parasite numbers in ears of infected mice [42].

We studied susceptibility to *L. tropica* using BALB/c-c-STS/A (CcS/Dem) recombinant congenic (RC) strains [43], which differ greatly in susceptibility to *L. major* [44], [45]. Parental strains BALB/c, STS and RC strains CcS-3, CcS-5, CcS-11, CcS-12, CcS-16, CcS-18, and CcS-20 were infected with *L. tropica* and skin lesions, cytokine and chemokine levels in serum, splenomegaly, hepatomegaly, and parasite numbers in organs were measured [46]. These experiments revealed that manifestations of the disease after infection with *L. tropica* are strongly influenced by genotype of the host. We have found that females of the RC strain CcS-16 that contains 12.5% genes of the resistant donor strain STS and 87.5% genes of the susceptible strain BALB/c [43], [47] developed the largest skin lesions and exhibited a unique systemic chemokine reaction, characterized by additional transient early peaks of CCL3 and CCL5, which were present neither in CcS-16 males nor in any other tested RC strain [46]. In order to establish the genetic basis of these differences, we prepared F_2_ hybrids between BALB/c and CcS-16, infected them with *L. tropica* and measured their skin lesions, splenomegaly, hepatomegaly, parasite numbers in spleen, liver and inguinal lymph nodes, and serum level of CCL3, CCL5 and CCL7 during the transient early peak. The strain CcS-16 carries STS-derived segments on nine chromosomes. They were genotyped in the F_2_ hybrid mice and their linkage with pathological symptoms and systemic immune responses was determined, which revealed eight controlling genes.

## Materials and Methods

### Mice

Females of strains BALB/c (16 infected, 16 uninfected) and CcS-16 (15 infected, 11 uninfected) were 8 to 19 weeks old (mean age 12 weeks, median age 12 weeks) at the time of infection. When used for these experiments, strain CcS-16 was in more than 90 generations of inbreeding. The parts of its genome inherited from the BALB/c or STS parents were defined [48]. 247 female F_2_ hybrids between CcS-16 and BALB/c (age 9 to 16 weeks at the time of infection, mean age 13 weeks, median 13 weeks) were produced at the Institute of Molecular Genetics AS CR, v.v.i.. Mice were kept in individually ventilated cages (Ehret, Emmendingen, Germany) and tested in two experimental groups. Both groups of F_2_ hybrids were derived from the same F_1_ parents; second experiment started seven weeks after the first. 2 mice died shortly after inoculation and were excluded from experiments. Among analyzed F_2_ hybrids, first experiment consisted of 111 mice, of which 51 mice originated from a cross (BALB/c×CcS-16)F_2_ (mean age 11.9 weeks, median 12 weeks; 3 mice died before the end of an experiment), 60 mice originated from a cross (CcS-16×BALB/c)F_2_ (mean age 12.6 weeks, median age 13 weeks; 1 mouse died before the end of an experiment). According to the nomenclature rules, the first strain listed in the cross symbol is the female parent, the second the male. The second experiment contained 134 mice, of which 64 mice originated from a cross (BALB/c×CcS-16)F_2_ (mean age 12.6 weeks, median 16 weeks; 2 mice died before the end of an experiment), 70 mice originated from a cross (CcS-16×BALB/c)F_2_ (mean age 13.4 weeks, median age 13 weeks; 6 mice died before the end of an experiment). The numbers of mice analyzed for individual phenotypes are given in Supplementary Table S1.

### Ethics statement

All experimental protocols utilized in this study comply with the Czech Government Requirements under the Policy of Animal Protection Law (No. 246/1992) and with the regulations of the Ministry of Agriculture of the Czech Republic (No. 207/2004), which are in agreement with all relevant European Union guidelines for work with animals and were approved by the Institutional Animal Care Committee of the Institute of Molecular Genetics AS CR and by Departmental Expert Committee for the Approval of Projects of Experiments on Animals of the Academy of Sciences of the Czech Republic (permission Nr. 37/2007).

### Parasite

*Leishmania tropica* from Urfa, Turkey (MHOM/1999/TR/SU23) was used for infecting mice. Amastigotes were transformed to promastigotes using SNB-9 [49], and 1×10^7^ stationary phase promastigotes from subculture 2 were inoculated in 50 µl of sterile Phosphate Buffer Saline (PBS) s.c. into the tail base, with promastigote secretory gel (PSG) collected from the midgut of *L. tropica*-infected *Phlebotomus sergenti* females (laboratory colony originating from *L. tropica* focus in Urfa). PSG was collected as described [50]. The amount corresponding to one sand fly female was used per mouse.

### Disease phenotype

The size of the skin lesions was measured every second week using the Profi LCD Electronic Digital Caliper Messschieber Schieblehre Messer (Shenzhen Xtension Technology Co., Ltd. Guangdong, China), which has accuracy 0.02 mm. Blood was collected every 2 weeks in volume from 60 to 180 µl, and serum was frozen at −30°C for further analysis. The mice were killed 43 weeks after inoculation. Blood, spleen, liver and inguinal lymph nodes were collected for later analysis.

### Quantification of parasite load

Parasite load was measured in frozen lymph nodes, spleen, and liver samples using PCR-ELISA according to the previously published protocol [51]. Briefly, total DNA was isolated using a TRI reagent (Molecular Research Center, Cincinnati, USA) standard procedure (http://www.mrcgene.com/tri.htm). For PCR, two primers (digoxigenin-labeled F 5′-ATT TTA CAC CAA CCC CCA GTT-3′ and biotin-labeled R 5′-GTG GGG GAG GGG CGT TCT-3′ (VBC Genomics Biosciences Research, Austria) were used for amplification of the 120-bp conservative region of the kinetoplast minicircle of *Leishmania* parasite, and 50 ng of extracted DNA was used per each PCR reaction. For a positive control, 20 ng of *L. tropica* DNA per reaction was amplified as a highest concentration of standard. A 30-cycle PCR reaction was used for quantification of parasites in lymph nodes; 33 cycles for spleen, and 40 cycles for liver. Parasite load was determined by analysis of the PCR product by the modified ELISA protocol (Pharmingen, San Diego, USA). Concentration of *Leishmania* DNA was determined using the ELISA Reader Tecan and the curve fitter program KIM-E (Schoeller Pharma, Prague, Czech Republic) with least squares-based linear regression analysis.

### Chemokines and cytokine levels

Levels of GM-CSF (granulocyte-macrophage colony-stimulating factor), CCL2 (chemokine ligand 2)/MCP-1 (monocyte chemotactic protein-1), CCL3/MIP-1α (macrophage inflammatory protein-1α), CCL4/MIP-1β (macrophage inflammatory protein-1β), CCL5/RANTES (regulated upon activation, normal T-cell expressed, and secreted) and CCL7/MCP-3 (monocyte chemotactic protein-3) in serum were determined using Mouse chemokine 6-plex kit (eBioscience, Vienna, Austria). The kit contains two sets of beads of different size internally dyed with different intensities of fluorescent dye. The set of small beads was used for GM-CSF, CCL5/RANTES and CCL4/MIP-1β and the set of large beads for CCL3/MIP-1α, CCL2/MCP-1 and CCL7/MCP-3. The beads are coated with antibodies specifically reacting with each of the analytes (chemokines) to be detected in the multiplex system. A biotin secondary antibody mixture binds to the analytes captured by the first antibody. Streptavidin-phycoerythrin binds to the biotin conjugate and emits a fluorescent signal. The test procedure was performed in the 96 well filter plates (Millipore, USA) according to the protocol of manufacturer. Beads were analyzed on flow cytometer LSR II (BD Biosciences, San Jose, USA). Lyophilized GM-CSF and chemokines (CCL2/MCP-1, CCL3/MIP1α, CCL4/MIP1β, CCL5/RANTES, CCL7/MCP-3) supplied in the kit were used as standards. Concentration was evaluated by Flow Cytomix Pro 2.4 software (eBioscience, Vienna, Austria). The limit of detection of each analyte was determined to be for GM-CSF 12.2 pg/ml, CCL2/MCP-1 42 pg/ml, CCL7/MCP-3 1.4 pg/ml, CCL3/MIP-1α 1.8 pg/ml, CCL4/MIP-1β 14.9 pg/ml, and for CCL5/RANTES 6.1 pg/ml.

### Genotyping of F_2_ mice

DNA was isolated from tails using a proteinase procedure [52] with modifications described in [51]. The strain CcS-16 differs from BALB/c at STS-derived regions on nine chromosomes [48 and unpublished results]. These differential regions were typed in the F_2_ hybrid mice between CcS-16 and BALB/c using 23 microsatellite markers (Generi Biotech, Hradec Králové, Czech Republic): D2Mit156, D2Mit389, D2Nds3, D2Mit257, D2Mit283, D2Mit52, D3Mit25, D3Mit11, D4Mit153, D6Mit48, D6Mit320, D10Mit67, D10Mit103, D11Mit139, D11Mit242, D11Nds18, D11Mit37, D16Mit126, D17Mit38, D17Mit130, D18Mit35, D18Mit40 and D18Mit49 (Supplementary Table S2). The maximum distance between any two markers in the chromosomal segments derived from the strain STS or from the nearest BALB/c derived markers was 14.16 cM. The DNA genotyping by PCR was performed as described elsewhere [53]. The genotyping for microsatellite markers with fragment length difference of less than 10 bp was performed by using ORIGINS Elchrom Scientific electrophoresis (Elchrom Scientific AG, Cham, Switzerland) according to manufacturer's instruction. Briefly, DNA was amplified as described in [53]. Each PCR product was mixed with 5 µl of loading buffer and electrophoresed using Spreadex EL300 gel and Spreadex EL400 gel (Elchrom Scientific AG, Cham, Switzerland) for product with size of less than 150 bp or more than 150 bp, respectively. The best gel and proper running time was selected using ElQuantTM Software (Elchrom Scientific AG, Cham, Switzerland). 30 mM TAE buffer was used as a running buffer. Running temperature was set to 20°C and to 50°C, when voltage was set to 120 V and 100 V, respectively. After finishing the electrophoresis gel was stained by ethidium bromide and the results were read by GENE bio-imaging system (Syngene, Cambridge, UK).

### Statistical analysis

The role of genetic factors in control of the tested pathological and immunological symptoms was examined with ANOVA using the program Statistica for Windows 8.0 (StatSoft, Inc., Tulsa, Oklahoma, USA). Marker, grandparent-of-origin effect and age were fixed factors and the experiment was considered as a random factor. In order to obtain normal distribution of the analyzed parameters, the obtained values were transformed, each as required by its distribution, as shown in the legends of the Tables. Markers and interactions with *P*<0.05 were combined in a single comparison.

To obtain whole genome significance values (corrected *P*-values) the observed *P*-values (αT) were adjusted according to Lander and Schork [54] using the formula:
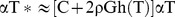
where G = 1.75 Morgan (the length of the segregating part of the genome: 12.5% of 14 M); C = 9 (number of chromosomes segregating in cross between CcS-16 and BALB/c, respectively); ρ = 1.5 for F_2_ hybrids; h(T) = the observed statistic (F ratio).

The percent of total phenotypic variance accounted for by a QTL and its interaction terms was computed by subtracting the sums of squares of the model without this variable from the sum of squares of the full model and this difference divided by the total regression sums of squares: 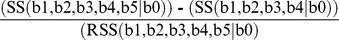



## Results

### Genetic control of skin lesions development

**Figure 1:**
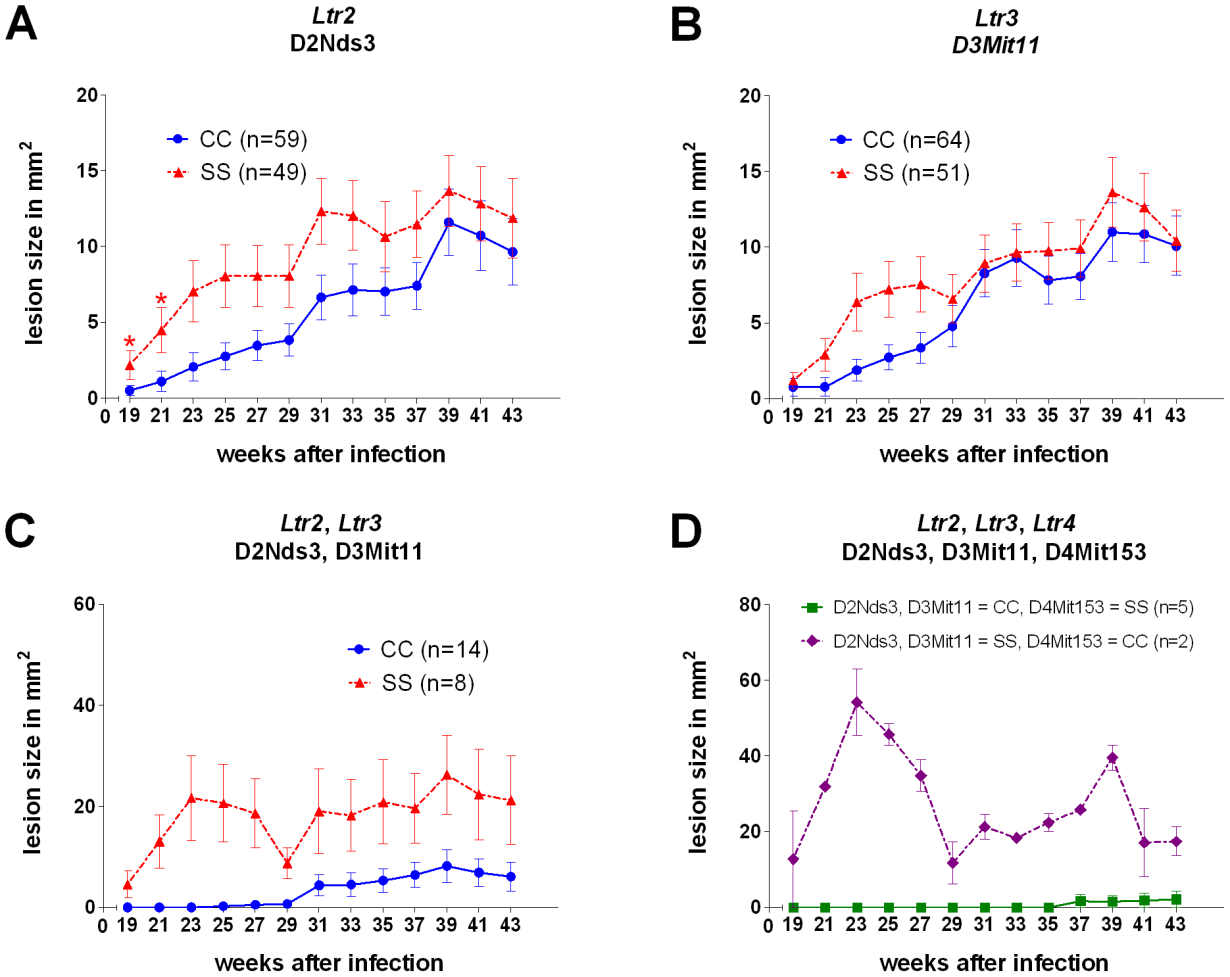
Differential lesion development in F_2_ hybrid mice carrying one, two and three *Ltr* loci after infection with *L. tropica*. **A.** F_2_ hybrids carrying BALB/c or STS homozygous (resistant or susceptible) alleles in *Ltr2* (D2Nds3); **B.** F_2_ hybrids carrying BALB/c or STS homozygous (resistant or susceptible) alleles in *Ltr3* (D3Mit11); **C.** F_2_ hybrids carrying BALB/c or STS homozygous (both resistant or both susceptible) alleles in both *Ltr2* (D2Nds3) and *Ltr3* (D3Mit11); **D.** F_2_ hybrids carrying BALB/c homozygous (both resistant) alleles in *Ltr2* and *Ltr3* and STS (resistant) homozygous alleles in *Ltr4* (D4Mit153), and F_2_ hybrids carrying STS homozygous (both susceptible) alleles in *Ltr2* and *Ltr3* and BALB/c (susceptible) homozygous alleles in *Ltr4*. n, number of mice. Graphs summarize data from two independent experimental groups and give non-normalized lesion sizes. Lesions were measured every second week. CC and SS indicate the homozygosity of BALB/c and STS allele, respectively. Please note different scales of Figures 1A,B, 1C and 1D.

Differences in skin lesions development between strains BALB/c and CcS-16 are controlled by two loci, which are not dependent on or influenced by interaction with other genes (main effects) (Table 1, Figure 1). *Ltr2* (*Leishmania tropica* response 2) linked to D2Nds3 (Figure 1A) and D2Mit389 influences lesion size at week 19 (corrected *P* = 0.004, Bonferroni corr. *P* = 0.049), 21 (corrected *P* = 0.0020, Bonferroni corr. *P* = 0.024) and 31 (corrected *P* = 0.0152, Bonferroni corr. *P* = 0.18) after infection, *Ltr3* that controls lesion size at week 21 after infection is linked to D3Mit11 (corrected *P* = 0.042, Bonferroni corr. *P* = 0.5) (Figure 1B). STS allele of both *Ltr2* and *Ltr3* determines larger lesions. STS allele of *Ltr4* marked by D4Mit153 (which also controls parasite numbers in liver and in lymph nodes) has an opposite effect on the studied trait; its STS allele is associated with smaller lesions at week 27 after infection. Figure 1C and Figure 1D show the strong additive effects of *Ltr2* and *Ltr3*, and *Ltr2*, *Ltr3* and *Ltr4*, respectively. However, *Ltr3* and *Ltr4* effects on skin lesions (nominal *P* value = 0.00048 and 0.00096, respectively, corr. *P* value = 0.024 and 0.045, respectively) were not significant after Bonferroni correction for number of tested weeks of infection and for whole genome significance. Although lesions were larger in the second experiment, no significant interaction between experimental group and markers was observed.

**Table 1:** Loci that control skin lesion development.

Phenotype	Locus	Marker	Genotype									*P* value	corr. *P* value	Bonfer. corr. *P* value	% of explained variance
			CC			CS			SS						
**lesion wk 19**	***Ltr2***	**D2Nds3**	**0.00**	0.002±0.001	(n = 60)	**0.35**	0.004±0.001	(n = 127)	**1.65**	0.010±0.001	(n = 53)	6.6×10^−5^	**0.004**	**0.049**	20.90
**lesion wk 19**	***Ltr4***	**D4Mit153**	**1.31**	0.008±0.001	(n = 54)	**0.23**	0.003±0.001	(n = 130)	**0.38**	0.004±0.001	(n = 56)	0.0018	0.077	0.93	9.66
**lesion wk 21**	***Ltr2***	**D2Nds3**	**0.20**	0.003±0.002	(n = 60)	**0.36**	0.004±0.001	(n = 127)	**2.27**	0.012±0.002	(n = 53)	2.9×10^−5^	**0.002**	**0.024**	10.93
**lesion wk 21**	***Ltr3***	**D3Mit11**	**0.39**	0.004±0.002	(n = 64)	**0.42**	0.004±0.001	(n = 122)	**1.94**	0.011±0.002	(n = 54)	0.00088	**0.042**	0.50	12.48
**lesion wk 23**	***Ltr3***	**D3Mit11**	**0.25**	0.149±0.010	(n = 64)	**0.28**	0.152±0.008	(n = 122)	**1.29**	0.203±0.012	(n = 54)	0.00053	**0.026**	0.32	6.59
**lesion wk 25**	***Ltr3***	**D3Mit11**	**0.52**	0.069±0.008	(n = 64)	**0.48**	0.067±0.007	(n = 122)	**2.21**	0.110±0.009	(n = 54)	0.00048	**0.024**	0.29	12.02
**lesion wk 27**	***Ltr4***	**D4Mit153**	**1.75**	0.101±0.008	(n = 54)	**0.59**	0.071±0.006	(n = 130)	**0.30**	0.060±0.008	(n = 55)	0.00096	**0.045**	0.54	7.53
**lesion wk 31**	***Ltr2***	**D2Mit389**	**1.32**	0.092±0.012	(n = 55)	**1.57**	0.097±0.007	(n = 134)	**5.53**	0.148±0.011	(n = 48)	0.00028	**0.015**	0.18	10.77

Lesions were measured every second week. In order to normalize distribution of the observed values, the natural logarithm of the lesion size (mm^2^) at each measured week (value+1.5) raised to the power of 0.04 was further raised to the power of 1.5 for weeks 19, 21, 23, 25; and to the power of 0.75 for week 31. The table shows means and SE calculated by analysis of variance. Non-transformed values of mean are given in bold. Number of tested mice is shown in brackets. Only *P* values significant after correction for genome-wide analysis and Bonferroni correction (multiplied by the number of tested weeks) are given. C and S indicate the presence of BALB/c and STS allele, respectively.

### Genetic control of parasite numbers in organs and visceral pathology

#### Parasite numbers in spleen and splenomegaly are controlled by different sets of genes

Parasite numbers in spleen are controlled by two loci (Table 2). STS allele of *Ltr3* linked with D3Mit25 (corrected *P* = 0.0085) determines lower parasite load, whereas STS allele of *Ltr6* (linked with D11Mit37) (corrected *P* = 0.014) is associated with higher parasite numbers. These *P*-values for *Ltr3* and *Ltr6* were significant only in cross (BALB/c×CcS-16)F_2_ (where mother of the F_1_ hybrids was BALB/c and father was CcS-16), but not in cross (CcS-16×BALB/c)F_2_ (where mother was CcS-16 and father was BALB/c). Interaction between the cross and marker D3Mit25 is highly significant (corr. *P* = 0.0013). Younger mice (from 9 to 12 weeks, mean = 11 weeks) have higher parasite load than the older (from 13 to 16 weeks, mean = 14 weeks) mice, but interaction between the marker and age was not significant (nominal *P* = 0.86).

**Table 2:** Main effect loci: control of parasite load in spleen and in liver, and visceral pathology.

Phenotype	Locus	Marker	Genotype									*P* value	corr. *P* value	% of expl. variance
			CC			CS			SS					
**Parasites in spleen**														
Both crosses	***Ltr3***	**D3Mit25**	**0.80**	4.38±0.16	(n = 61)	**0.63**	4.15±0.13	(n = 108)	**0.48**	3.87±0.17	(n = 62)	0.094	NS	NA
(BALB/c×CcS-16)F_2_	***Ltr3***	**D3Mit25**	**1.72**	5.15±0.22	(n = 29)	**0.75**	4.32±0.21	(n = 43)	**0.43**	3.76±0.22	(n = 37)	0.00014	**0.0085**	19.38
(CcS-16×BALB/c)F_2_	***Ltr3***	**D3Mit25**	**0.38**	3.63±0.21	(n = 32)	**0.49**	3.89±0.15	(n = 65)	**0.61**	4.11±0.23	(n = 25)	0.304	NS	NA
**Parasites in spleen**														
Both crosses	***Ltr6***	**D11Mit37**	**0.57**	4.04±0.16	(n = 65)	**0.46**	3.84±0.12	(n = 105)	**0.96**	4.56±0.16	(n = 62)	0.0028	0.113	NA
(BALB/c×CcS-16)F_2_	***Ltr6***	**D11Mit37**	**0.65**	4.17±0.24	(n = 31)	**0.45**	3.81±0.21	(n = 46)	**1.75**	5.17±0.23	(n = 32)	0.00024	**0.014**	29.58
**Splenomegaly**	***Ltr8***	**D18Mit49**	**5.28**	1.70±0.06	(n = 74)	**4.67**	1.57±0.05	(n = 106)	**3.60**	1.30±0.07	(n = 53)	0.00022	**0.012**	18.59
**Parasites in liver**	***Ltr2***	**D2Nds3**	**0.61**	4.12±0.11	(n = 60)	**0.83**	4.42±0.08	(n = 123)	**1.25**	4.83±0.14	(n = 49)	0.00056	**0.028**	9.50
**Hepatomegaly**	***Ltr2***	**D2Mit389**	**45.76**	37.41±0.81	(n = 55)	**42.28**	34.66±0.52	(n = 131)	**48.31**	39.42±0.86	(n = 46)	4.3×10^−6^	**0.00033**	13.83

Parasite numbers (week 43) were estimated by PCR–ELISA. Means, SE and *P* values for splenomegaly (week 43), hepatomegaly (week 43) and concentration of parasite DNA (ng/µl) in isolates from lymph nodes, spleen and liver were calculated by analysis of variance. Normal distribution was obtained for splenomegaly (spleen-to-body weight ratio×1000) by raising values to the power of 0.00002. Hepatomegaly (liver-to-body weight ratio×1000) was normalized by raising values to the power of 0.0125. To obtain normal distribution for parasite load in organs, the following transformations were used: natural logarithm of (value×100). The numbers in bold give the average non-transformed values. Only *P* values significant after correction for genome-wide testing are given. Number of tested mice is shown in brackets. C and S indicate the presence of BALB/c and STS allele, respectively. NS – not significant, NA – not applicable.

Splenomegaly is controlled by five loci (Table 2, 3). *Ltr8* linked with D18Mit49 (corr. *P* = 0.012) has a main effect, its BALB/c allele is associated with a larger spleen to body weight ratio. *Ltr2*, *Ltr3*, *Ltr5* and *Ltr7* affect splenomegaly in gene-gene interactions. *Ltr2* linked to D2Mit257 influences splenomegaly in interaction with *Ltr3* linked to D3Mit11 (corrected *P* = 0.010). F_2_ mice with homozygous STS (SS) alleles at both *Ltr2* and *Ltr3* have the smallest splenomegaly. *Ltr5* linked to D10Mit103 influences splenomegaly in interaction with *Ltr8* linked to D18Mit49 (corrected *P* = 0.029). F_2_ mice with homozygous STS (SS) alleles at both *Ltr5* and *Ltr8* have the smallest splenomegaly. *Ltr5* also influences splenomegaly in interaction with *Ltr7* linked to D17Mit30 (corrected *P* = 0.029). F_2_ mice with homozygous BALB/c (CC) alleles at *Ltr5* and homozygous STS (SS) alleles at *Ltr7* have the most severe splenomegaly, the other genotypes show no pronounced differences.

**Table 3:** Interaction between loci that control splenomegaly after 43 weeks of *L. tropica* infection.

				*P* = 0.00026		Corrected *P* = 0.010		% of explained variance = 9.05
		D2Mit257 (*Ltr2*)						
		CC		CS		SS		
**D3Mit11**	CC	**3.98**	1.41±0.09	**4.58**	1.55±0.06	**5.24**	1.69±0.08	
**(** ***Ltr3*** **)**			(n = 14)		(n = 33)		(n = 17)	
	CS	**4.51**	1.54±0.06	**4.35**	1.50±0.04	**5.32**	1.71±0.08	
			(n = 36)		(n = 63)		(n = 19)	
	SS	**4.37**	1.50±0.10	**5.23**	1.69±0.06	**3.12**	1.16±0.13	
			(n = 13)		(n = 31)		(n = 7)	

				*P* = 0.00083		Corrected *P* = 0.029		% of explained variance = 6.78
		D10Mit103 (*Ltr5*)						
		CC		CS		SS		
**D18Mit49**	CC	**5.03**	1.65±0.10	**5.03**	1.65±0.07	**5.81**	1.80±0.10	
**(** ***Ltr8*** **)**			(n = 17)		(n = 34)		(n = 23)	
	CS	**4.50**	1.53±0.08	**4.51**	1.54±0.07	**5.03**	1.65±0.07	
			(n = 28)		(n = 45)		(n = 33)	
	SS	**4.11**	1.44±0.12	**4.38**	1.51±0.08	**2.60**	0.97±.12	
			(n = 12)		(n = 32)		(n = 9)	

				*P* = 0.00083		Corrected *P* = 0.029		% of explained variance = 7.54
		D10Mit67 (*Ltr5*)						
		CC		CS		SS		
**D17Mit130**	CC	**4.73**	1.59±0.10	**4.15**	1.45±0.07	**4.67**	1.57±0.09	
**(** ***Ltr7*** **)**			(n = 14)		(n = 32)		(n = 16)	
	CS	**4.36**	1.50±0.06	**5.06**	1.66±0.05	**4.29**	1.48±0.06	
			(n = 35)		(n = 62)		(n = 28)	
	SS	**6.11**	1.85±0.09	**4.07**	1.43±.09	**4.65**	1.57±0.10	
			(n = 16)		(n = 18)		(n = 12)	

Means, SE and *P* values for splenomegaly were calculated by analysis of variance. Normal distribution was obtained for splenomegaly (spleen-to-body weight ratio×1000) by raising values to the power of 0.00002. The numbers in bold give the average non-transformed values. Only *P* values significant after correction for genome-wide significance are given. Number of tested mice is shown in brackets. C and S indicate the presence of BALB/c and STS allele, respectively.

#### Parasite numbers in liver are controlled by *Ltr2*, *Ltr4* and *Ltr8*, whereas hepatomegaly is influenced by *Ltr2* only

Parasite numbers in liver are controlled by three genes (Table 2, 4). *Ltr2* linked to D2Nds3 (corrected *P* = 0.028) has a main effect on parasite numbers in liver. Its STS allele is associated with a higher parasite load (Table 2). *Ltr4* linked to D4Mit153 influences parasite load in liver in interaction with *Ltr8* linked to D18Mit40 (corrected *P* = 0.021). F_2_ mice with homozygous BALB/c (CC) alleles at *Ltr4* and heterozygous at *Ltr8* have the highest parasite burden in liver.

**Table 4:** Interaction between loci controlling parasite burden in lymph nodes and liver 43 weeks after infection.

Interaction between loci that control parasite burden in liver								
				*P* = 0.00059		Corrected *P* = 0.021		% of explained variance = 8.11
		D18Mit40 (*Ltr8*)						
		CC		CS		SS		
**D4Mit153**	CC	**0.85**	4.44±0.2	**1.19**	4.78±0.2	**0.51**	3.93±0.21	
**(** ***Ltr4*** **)**			(n = 14)		(n = 25)		(n = 14)	
	CS	**1.00**	4.61±0.14	**0.65**	4.17±0.09	**1.05**	4.65±0.18	
			(n = 33)		(n = 72)		(n = 19)	
	SS	**0.67**	4.21±0.2	**0.73**	4.28±0.18	**0.85**	4.44±0.20	
			(n = 13)		(n = 22)		(n = 20)	

Interaction between loci that control parasite burden in inguinal lymph nodes								
				*P* = 0.00094		Corrected *P* = 0.032		% of explained variance = 7.31
		D4Mit153 (*Ltr4*)						
		CC		CS		SS		
**D2Mit156**	CC	**0.87**	4.46±0.32	**0.73**	4.29±0.25	**3.86**	5.96±0.45	
**(** ***Ltr1*** **)**			(n = 17)		(n = 26)		(n = 8)	
	CS	**0.74**	4.31±0.25	**0.67**	4.21±0.17	**0.50**	3.91±0.23	
			(n = 26)		(n = 64)		(n = 31)	
	SS	**1.78**	5.18±0.40	**0.71**	4.26±0.23	**0.34**	3.54±0.38	
			(n = 10)		(n = 32)		(n = 12)	

Means, SE and *P* values for concentration of parasite DNA (ng/µl) in isolates from lymph nodes and liver were computed by analysis of variance. The following transformations were used to obtain normal distribution: natural logarithm of (value×100). Hepatomegaly (liver-to-body weight ratio×1000) was normalized by raising values to the power of 0.0125. The numbers in bold give the average non-transformed values. Only *P* values significant after correction for genome-wide significance are given. Number of tested mice is shown in brackets. C and S indicate the presence of BALB/c and STS allele, respectively.

Hepatomegaly is determined by locus *Ltr2* linked to D2Mit389 (corrected *P* = 0.00033) (Table 2). Less severe hepatomegaly was observed in heterozygotes.

#### Genetic control of parasite load in inguinal lymph nodes

Parasite numbers in inguinal lymph nodes are influenced by interaction between *Ltr1* linked to D2Mit156 and *Ltr4* linked to D4Mit153 (corrected *P* = 0.032). Highest parasite load is observed in F_2_ mice with homozygous STS (SS) alleles at *Ltr4* and homozygous BALB/c (CC) alleles at *Ltr1* (Table 4). There was no interaction between experimental group and markers (nominal *P* = 0.89).

### Genetic control of early peak of chemokines level in serum of infected mice

Genetic analysis of F_2_ hybrids has revealed identical genetic control of serum levels of CCL3 and CCL5 at week 7 after infection (Table 5, 6). *Ltr3* linked to D3Mit11 determines levels of both CCL3 (corrected *P* = 0.0046) and CCL5 (corrected *P* = 0.010), its BALB/c allele is associated with higher chemokine levels (Table 5). *Ltr3* has not only individual (main) effect on chemokines levels, but also influences levels of CCL3 (corrected *P* = 0.014) and CCL5 (corrected *P* = 0.0012) in interaction with *Ltr7* linked to D17Mit130. The largest effect is seen by *Ltr3* when *Ltr7* is SS. In that genotypic situation the *Ltr3* CC alleles cause more than 300×higher levels of CCL3 and 28×higher levels of CCL5 than the *Ltr3* SS alleles (Table 6). It is likely that this very large size of this effect in *Ltr7* SS mice makes the *Ltr3* effects visible as a main effect, although smaller, in F_2_ hybrids irrespective of their *Ltr7* genotype.

**Table 5:** Main effect of loci controlling serum chemokine level after 7 weeks of infection.

Phenotype	Locus	Marker	Genotype									*P* value	corr. *P* value	% of explained variance
			CC			CS			SS					
**CCL3**	***Ltr3***	**D3Mit11**	**711.42**	3.72±0.18	(n = 64)	**371.57**	3.27±0.12	(n = 118)	**94.68**	2.49±0.21	(n = 53)	7.5×10^−5^	**0.0046**	4.56
**CCL5**	***Ltr3***	**D3Mit11**	**2724.44**	5.15±0.08	(n = 64)	**1805.94**	4.98±0.05	(n = 117)	**861.34**	4.66±0.09	(n = 53)	0.00018	**0.010**	3.99
**CCL7**	***Ltr2***	**D2M52**	**566.41**	6.34±0.05	(n = 48)	**590.60**	6.38±0.03	(n = 127)	**740.99**	6.61±0.05	(n = 60)	3×10^−5^	**0.002**	9.06
**CCL7**	***Ltr8***	**D18M40**	**766.86**	6.64±0.05	(n = 60)	**602.67**	6.40±0.04	(n = 118)	**613.11**	6.42±0.06	(n = 55)	0.00024	**0.013**	11.38

In order to normalize distribution of the observed values (in pg/ml), the following transformations were used: the power of 0.2 (concentration value+1) – CCL3/MIP1α; natural logarithm – CCL7/MCP-3; the power of −0.117545 followed by subtraction with 1 – CCL5/RANTES. In case of CCL5/RANTES, the calculated value was further divided by −0.117545. The Table gives mean of non-transformed (in bold) and transformed concentration and SE of the transformed values calculated by analysis of variance. Only *P* values significant after correction for genome-wide significance are given. Number of tested mice is shown in brackets. C and S indicate the presence of BALB/c and STS allele, respectively.

**Table 6:** Interaction between loci that control chemokines level after 7 weeks of *L. tropica* infection.

A. CCL3/MIP-1α				*P* = 0.00036		Corrected *P* = 0.014		% of variance = 3.33
		D3Mit11 (*Ltr3*)						
		CC		CS		SS		
**D17Mit130 (** ***Ltr7*** **)**	CC	**298.60**	3.13±0.36	**377.81**	3.28±0.22	**172.79**	2.81±0.30	
			(n = 12)		(n = 32)		(n = 17)	
	CS	**358.96**	3.25±0.20	**248.17**	3.02±0.17	**288.44**	3.11±0.23	
			(n = 40)		(n = 58)		(n = 30)	
	SS	**2511.51**	4.79±0.36	**531.34**	3.51±0.24	**8.15**	1.56±0.51	
			(n = 12)		(n = 28)		(n = 6)	

B. CCL5/RANTES				*P* = 2.7×10^−5^		Corrected *P* = 0.0012		% of variance = 3.96
		D3Mit11 (*Ltr3*)						
		CC		CS		SS		
**D17Mit130 (** ***Ltr7*** **)**	CC	**1730.31**	4.97±0.15	**2023.41**	5.03±0.10	**2298.59**	5.08±0.14	
			(n = 12)		(n = 32)		(n = 17)	
	CS	**1977.17**	5.02±0.08	**1616.45**	4.94±0.07	**2338.11**	5.09±0.11	
			(n = 40)		(n = 57)		(n = 30)	
	SS	**7542.52**	5.53±0.15	**2144.55**	5.05±0.10	**272.74**	4.11±0.22	
			(n = 12)		(n = 28)		(n = 6)	

C. CCL7/MCP-3				*P* = 0.00044		Corrected *P* = 0.016		% of variance = 6.66
		D2Mit257 (*Ltr2*)						
		CC		CS		SS		
**D11Mit37 (** ***Ltr6*** **)**	CC	**579.33**	6.36±0.08	**573.80**	6.35±0.05	**547.09**	6.30±0.10	
			(n = 17)		(n = 36)		(n = 10)	
	CS	**693.53**	6.54±0.07	**585.28**	6.37±0.04	**683.23**	6.53±0.07	
			(n = 30)		(n = 58)		(n = 22)	
	SS	**513.90**	6.24±0.08	**736.45**	6.60±0.06	**725.32**	6.59±0.09	
			(n = 17)		(n = 32)		(n = 13)	

In order to normalize distribution of the observed values, the concentration in pg/ml was raised to the power of 0.2 (concentration value+1) – CCL3/MIP1α; to the power of −0.117545 followed by subtraction with 1 – CCL5/RANTES. In case of CCL5/RANTES, the calculated value was further divided by −0.117545. The Table gives mean of non-transformed (in bold) and transformed concentration and SE of the transformed values calculated by analysis of variance. Only *P* values significant after correction for genome-wide significance are given. Number of tested mice is shown in brackets. C and S indicate the presence of BALB/c and STS allele, respectively.

CCL7 level is controlled with two loci with an opposite effect on the studied trait. The homozygosity for the STS allele of *Ltr2* (SS) determines higher CCL7 level (corrected *P* = 0.002), whereas homozygosity for the BALB/c allele of *Ltr8* (CC) is associated with higher level of this chemokine (corrected *P* = 0.013) (Table 5). No significant interaction between experimental group and marker was observed. Older mice had higher levels of CCL7 in serum than the younger ones, but we did not observe any interactions between marker and age (nominal *P* (*Ltr2*) = 0.80, nominal *P* (*Ltr8*) = 0.64). Levels of CCL7 in serum of infected mice are also influenced by interaction of *Ltr2* linked to D2Mit257 and *Ltr6* linked to D11Mit37 (corrected *P* = 0.016), the highest CCL7 levels are observed in STS allele (SS) homozygotes in *Ltr6* in combination with heterozygotes (CS) or STS allele (SS) homozygotes in *Ltr2* (Table 6).

Although chemokine levels were higher in the first experiment, no significant interaction between experimental group and markers was observed.

No linkage was found for GM-CSF, CCL2/MCP-1 and CCL4/MIP-1β.

## Discussion

**Figure 2:**
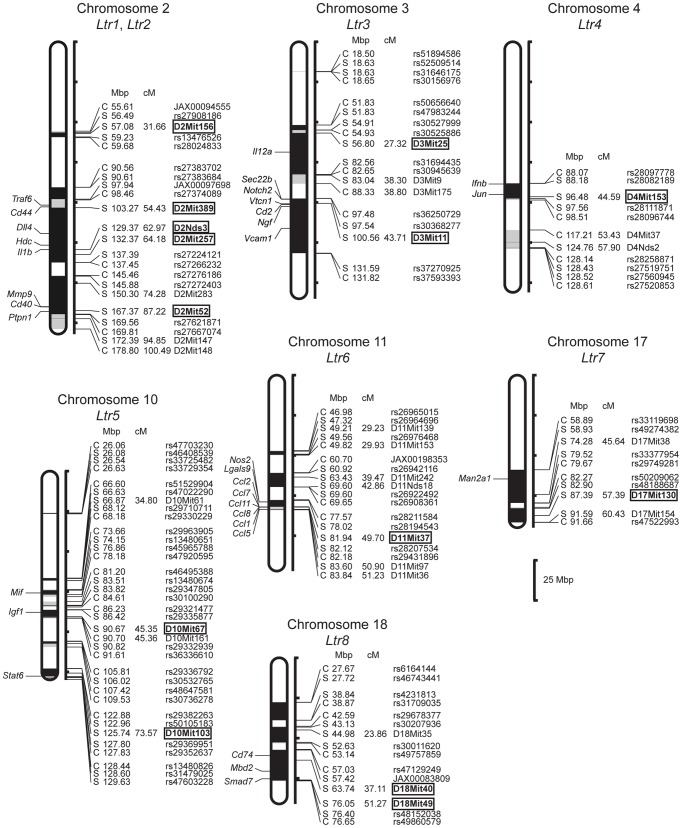
Position of the loci that control response to *L. tropica* in strain CcS-16. The regions of STS and BALB/c origin are represented as dark and white, respectively; the boundary regions of undetermined origin are shaded. Only the markers and SNPs defining the boundaries the STS-derived segment and the markers that were tested for linkage are shown. The markers that exhibit significant *P* values (corrected for genome-wide search) are shown in bold. Abbreviations show genes that have been reported to be involved in response to *Leishmania* ssp.: *Ccl1* (chemokine (C-C motif) ligand 1) [55], *Ccl11* (chemokine (C-C motif) ligand 11) [56], *Ccl2* (chemokine (C-C motif) ligand 2), *Ccl5* (chemokine (C-C motif) ligand 5) [57], *Ccl7* [58], *Cd2* (CD2 antigen) [59], *Cd40* (CD40 antigen) [60], *Cd44* (CD44 antigen) [61], *Cd74* (CD74 antigen) [62], *Dll4* (Delta-like 4) [63], *Hdc* (histidine decarboxylase) [64], *Ifnb1* (interferon beta 1) [65], *Igf1* (insulin-like growth factor 1) [66], *Il1* (interleukin 1) [67], *Il12a* (Interleukin 12a) [68], *Jun* (Jun oncogene) [69], *Lgals9* (lectin, galactose binding, soluble 9) [70], *Man2a1* (mannosidase 2, alpha 1) [71], *Mbd2* (methyl-CpG binding domain protein 2) [72], *Mif* (macrophage inhibitory factor) [73], *Mmp9* (matrix metalopeptidase 9) [74], *Ngf* (nerve growth factor) [75], *Nos2* (nitric oxide synthase 2, inducible) [76], *Notch2* (notch 2) [77], *Ptpn1* (protein tyrosine phosphatase, non-receptor type 1) [78], *Sec22b* (SEC22 vesicle trafficking protein homolog B (*S. cerevisiae*)) [79], *Smad7* (SMAD family member 7) [80], *Stat6* (Signal transducer and activator of transcription-6) [81], *Traf6* (TNF receptor associated factor 6) [60], *Vcam1* (vascular cell adhesion molecule 1) [82], *Vtcn1* (V-set domain containing T cell activation inhibitor 1) [83]. (Genes IDs are shown in Supplementary Table S3).

The present study provides the first insight into the genetic architecture of susceptibility to *L. tropica*. We have described eight loci on seven chromosomes (Figure 2 [10], [12], [55]–[83]) and shown that the presence of individual symptoms of disease is controlled by different subsets of host's genes. The identification of host's genes responsible for the specific symptoms of the disease induced by different *Leishmania* species will contribute to the understanding of mechanisms of pathogenesis of leishmaniasis, similarly as comparative parasite genomics led to identification of differentially distributed genes in *Leishmania* species inducing different pathology [84], [85], and analysis of specific virulence factors revealed how different *Leishmania* species subvert or circumvent host's defenses [7]. Such analysis will provide description of individual predisposition to specific symptoms of disease and its probable course. Moreover, the possibility to compare genetics of response to several *Leishmania* species will further help to understand the genetic basis of general and species-specific responses of the host. This will synergize with the future information about genome sequence of *L. tropica* and about interaction of its specific virulence factors with the immune system.

### Response to *L. tropica* is controlled by multiple genes with heterogeneous effects

**Figure 3:**
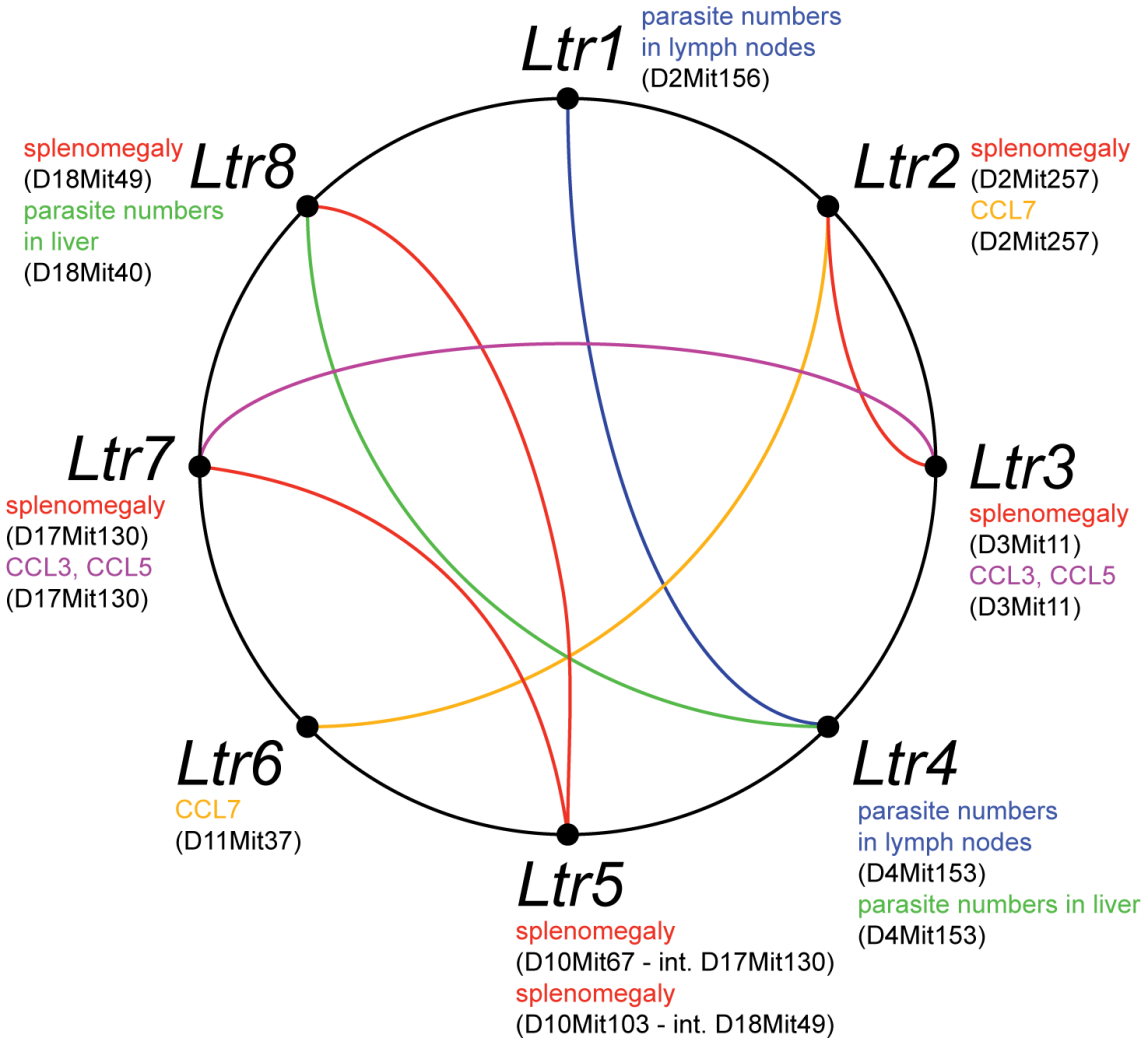
Interactions among loci that control response to *L. tropica*. Phenotypes controlled by each locus are shown at its symbol in different colors. The colored lines connecting the loci indicate interactions controlling the specific phenotypes.

Our data show that interaction of mice with *L. tropica* parasites is complex and involves numerous genes and responses (Table 7). We have detected eight loci that in the strain CcS-16 control host-parasite interaction (Table 7, Figure 2). All eight *Ltr* loci are involved in gene-gene interactions (Figure 3), four loci (*Ltr2*, *Ltr3*, *Ltr6*, *Ltr8*) have also individual effect, while effects of *Ltr1*, *Ltr4*, *Ltr5* and *Ltr7* are seen only in interaction with other *Ltr* loci. This is not surprising, as the average proportion of genetic variation explained by epistatic QTLs in mice in different systems was estimated to be 49% [86] and gene-gene interactions were observed also in response to other pathogens such as *L. major* [87]–[89], *Trypanosoma brucei brucei* [53], *Salmonella enteritidis* [90], *Plasmodium falciparum* [91] and *Mycobacterium leprae* [92].

**Table 7:** Summary of loci that control response to *L. tropica.*

chr.	locus	marker	Phenotype controlled
2	*Ltr1*	D2Mit156	parasites in lymph nodes (int. *Ltr4 -* D4Mit153)
2	*Ltr2*	D2Mit389; D2Nds3/*Il1b*; D2Mit257; D2Mit52	skin lesions wk 19; skin lesions wk 21; splenomegaly (int. *Ltr3* - D3Mit11); parasites in liver; hepatomegaly; CCL7; CCL7 (int. *Ltr6* - D11Mit37)
3	*Ltr3*	D3Mit25; D3Mit11	splenomegaly (int. *Ltr2* - D2Mit257); parasites in spleen (transgenerational parental effect); CCL3; CCL3 (int. *Ltr7* - D17Mit130); CCL5; CCL5 (int. *Ltr7* - D17Mit130)
4	*Ltr4*	D4Mit153	parasites in lymph nodes (int. *Ltr1* - D2Mit156); parasites in liver (int. *Ltr8* - D18Mit40)
10	*Ltr5*	D10Mit67; D10Mit103	splenomegaly (int. *Ltr7* - D17Mit130); splenomegaly (int. *Ltr8* - D18Mit49)
11	*Ltr6*	D11Mit37	parasites in spleen (transgenerational parental effect); CCL7 (int. *Ltr2* - D2Mit257)
17	*Ltr7*	D17Mit130	splenomegaly (int. *Ltr5* - D10Mit67); CCL3 (int. *Ltr3* - D3Mit11); CCL5 (int. *Ltr3* - D3Mit11)
18	*Ltr8*	D18Mit40; D18Mit49	splenomegaly; splenomegaly (int. *Ltr5* - D10Mit103); parasites in liver (int. *Ltr4* - D4Mit153); CCL7

The loci described here have heterogeneous effects (Table 7). *Ltr1* on chromosome 2 controls in interaction with *Ltr4* only parasite numbers in lymph nodes, whereas the more distal *Ltr2* on the same chromosome influences development of skin lesions, splenomegaly (in interaction with *Ltr3*), hepatomegaly, parasite load in liver and level of CCL7 in serum. Multiple functions are also exerted by *Ltr3* on chromosome 3, which controls splenomegaly (in interaction with *Ltr2*), parasite numbers in spleen, and levels of CCL3 and CCL5 in serum. We have analyzed genetic control of early levels of chemokines, as there is a unique early peak in the CcS-16 females [46]. However, comparison of genetic control of CCL3 and CCL5 levels with genetic control of development of skin lesions indicates that there is no simple correlation between the chemokines levels and manifestations of disease. *Ltr4* on chromosome 4 controls in interaction with *Ltr1* and *Ltr8* parasite numbers in lymph nodes and in liver, respectively. *Ltr5* on chromosome 10 influences in interaction with *Ltr7* or *Ltr8* splenomegaly. *Ltr6* influences parasite numbers in spleens and level of CCL7 in serum (in interaction with *Ltr2*). *Ltr7* controls splenomegaly (in interaction with *Ltr5*) and in interaction with *Ltr3* level of both CCL3 and CCL5 in serum. *Ltr8* controls splenomegaly (as a main effect gene and in interaction with *Ltr5*), parasite numbers in liver (in interaction with *Ltr4*) and level of CCL7 in serum. *Ltr1* and *Ltr5* control only one parameter, whereas other loci have multiple effects. Some multiple effects could reflect causal relationship – e.g. CCL7 influences recruitment of monocytes to spleen [93], which could contribute to splenomegaly. The observed multiple effects of some *Ltr* loci might also suggest that some such loci might represent complexes of two or more closely linked *Ltr* genes. This issue will be resolved by future recombinational analysis.

We have detected also loci that control symptoms, such as splenomegaly, in which the strains BALB/c and CcS-16 do not differ [46]. This is because in an inbred strain the final outcome of response is exerted by multiple genes, which often have opposite effects, masking each other. In the F_2_ hybrids these genes segregate and can be therefore detected.

Reliability and validity of the described loci is supported by the fact that they have been detected by analysis of different phenotypes and their statistical significance was corrected for whole genome testing and where appropriate also by conservative Bonferroni correction. The relatively high proportion of variance explained by the mapped loci (Table 1–6) might be partly due to a limited variability of the tested manifestations of the disease.

### Susceptibility alleles carried by a resistant strain

Most inbred mouse strains that were produced without intentionally selectively bred for a specific quantitative phenotype (like susceptibility to specific infections) inherited from their non-inbred ancestors randomly susceptible alleles at some loci and resistant alleles at others, so that their overall susceptibility phenotype depends on the relative number of both. STS is resistant to *L. tropica* and does not develop skin lesions [24], however some STS-derived segments carried by CcS-16 on chromosome 2 (*Ltr2*) and possibly also on chromosome 3 (*Ltr3*) are associated with larger lesions. Similarly, STS-derived alleles of *Ltr2* and *Ltr6* are associated with higher parasite load in liver and spleen, respectively. This finding is not unique as susceptibility alleles originating from resistant strains were found in studies of colon cancer [94] and *L. major* [95] susceptibility; a low-responder allele was identified in a strain exhibiting high response to IL-2 [96] or producing a high level of IFNγ [97], whereas a high responder allele was found in a strain producing low level of IL-4 [98].

### Transgenerational parental effect

Loci *Ltr3* and *Ltr6* influencing parasite numbers in spleen (Table 2) were significant only in the cross (BALB/c×CcS-16)F_2_, but not in the cross (CcS-16×BALB/c)F_2_, hence the outcome in these crosses that are theoretically genetically identical depends on the strain of the female or male used originally to produce the F_1_ hybrids, which were then crossed with each other to produce the F_2_ hybrids for the tests. Thus, this is a special type of a transgenerational parental effect as the mothers and fathers of the F_2_ hybrids were genetically identical. Recently, examples of transgenerational parental effects have been described in several species [reviewed in [99]] and several possible mechanisms have been proposed. Our observation may reflect a parental effect due to modification of the developing immune system of fetuses or youngs by maternal environment, maternal nutritional effects, or epigenetic effects, and it offers a possibility to characterize the transgenerationally regulated functional pathways.

### Control of parasite load is predominantly organ specific

Control of parasite elimination differs among organs: the loci *Ltr1* and *Ltr4* interact to control parasite numbers in inguinal lymph nodes, while *Ltr4* in interaction with *Ltr8* influences parasite load in liver (Table 4). Parasite load in liver is also controlled by *Ltr2* (Table 2), whereas parasite burden in spleen is influenced by *Ltr3* and *Ltr6* (Table 2). These data show that parasite elimination in lymph nodes, liver and spleen are controlled differently, suggesting a predominantly organ specific control of parasite load. Mechanistic studies analyzing response to *L. tropica* in different organs are not yet available, but generally organ specific responses described here are compatible with the mechanistic studies of other parasites. The enzymes inducible nitric oxide synthase and phagocyte NADPH oxidase, which are required for the control of *L. major*, display organ- and stage-specific anti-*Leishmania* effects [76], [100]. Inducible nitric oxide synthase has been shown to control resistance to parasites in skin and draining lymph nodes, but not in spleen of the resistant strain C57BL/6 [100]. On the other hand, activity of phagocyte NADPH oxidase is essential for the clearance of *L. major* in the spleen, but it is dispensable for the resolution of the acute skin lesions and it exerted only a limited effect on the containment of the parasites in the draining lymph node [76]. Similarly, *bg*/*Lyst* (lysosomal trafficking regulator) is involved in control of parasite numbers of *L. donovani* in spleen, but not in liver [31]. On the other hand VCAM-1 (vascular cell adhesion molecule-1) and VLA-4 (very late antigen-4) interactions influenced early *L. donovani* burden in liver, but not in spleen [82].

### Different control of parasite elimination and organ pathology

Comparison of genetic control of parasite numbers in spleen and splenomegaly, or parasite numbers in liver and hepatomegaly shows that control of parasites elimination and organ pathology overlap only partially. For example *Ltr3* controls both parasite numbers in spleen and splenomegaly, but *Ltr6* is involved in control of parasite numbers in spleen, but not in splenomegaly, whereas *Ltr2*, *Ltr8*, *Ltr5*, and *Ltr7* are involved only in control of splenomegaly (Table 2, 3, 7). Similarly, *Ltr2* influences both parasite load in liver and hepatomegaly, but parasite load in liver is controlled also by interaction of *Ltr4* with *Ltr8*. The differences in genetic control of parasite numbers and organ pathology induced by the parasites are probably due to the fact that during a chronic disease the organ damage is a combined result of speed of elimination of parasite on one hand and changes caused by reaction to parasite (such as influx of immune cells, inflammatory responses) and healing processes on the other hand. It is therefore likely that these processes are regulated by different sets of genes.

It is important to understand that as in any QTL study, failure to find a linkage between a phenotype and a marker does not rule out that such linkage may exist, although its phenotypic effect are likely smaller than in the detected linkages. So for a QTL, which affects several but not all parameters of a complex disease, this indicates that it has predominant effects on some parameters, although it might modify to a lesser extent other parameters as well.

### Comparison of genetic control of response to several pathogens

#### Comparison of loci that control response to *L. tropica* and *L. major* – indication of common and species-specific genes

Comparison of genetic control of response to *L. tropica* and *L. major* might indicate some common and some distinct mechanisms in response to these two parasites. We compared genetic relationship between the *Ltr* (this study) and *Lmr* [88], [95], [101] loci detected in the strain CcS-16. Loci *Ltr1* (chromosome 2), *Ltr4* (chromosome 4) and *Ltr7* (chromosome 17) appear to be species-specific and do not overlap with loci controlling response to *L. major*. *Ltr2* (chromosome 2) co-localizes with *Lmr14*, *Ltr5* (chromosome 10) with *Lmr5*, *Ltr6* (chromosome 11) with *Lmr15*, and *Ltr8* (chromosome 18) with *Lmr13*. *Ltr2* controls visceral pathology in both species and is also involved in additional responses, which are unique for each parasite. Moreover, *Ltr2* and *Lmr14* overlap with *Ir2*, which controls visceral pathology after infection with *L. donovani* [4]. The other co-localizing loci also influence different sets of symptoms and are often involved in different interactions. This might indicate either the presence of the same controlling genes, which function differently under exposure to *L. tropica* and *L. major*, or less likely, a chance coincidence – presence of different controlling genes on the same chromosomal segment.

*Ltr3* on chromosome 3 co-localizes with *Lmr11*, which was detected in the strain CcS-20, but not in the CcS-16, and which exhibits a single gene effect on IL-6 level in serum [88] and in interaction with *Lmr8* on chromosome 1 influences serum IgE level in *L. major*-infected mice [101].

#### Some loci affect susceptibility to several pathogens

Some loci affect responses to a very broad spectrum of pathogens. For example, locus *Ltr2* co-localizes also with *Bb15*, which controls specific and total IgG in serum after infection with *Borrelia burgdorferi* [102]. The most obvious potential candidate gene in this chromosomal segment is *Il1* (interleukin 1). IL-1β was found to be up-regulated in dermal lesions of patients with cutaneous leishmaniasis caused by *L. tropica* and decreased after therapy [103], IL-1 was also found to regulate visceral manifestation of murine leishmaniasis after infection with *L. major* [67], and polymorphism in *IL1B* was linked with disease severity in patients infected with *L. mexicana* [10]. IL-1 was also described to influence IgG level in autoimmunity [104], which might suggest its involvement in response to *B. burgdorferi*.

### Potential candidate genes

Usually, a standard inbred-strain mapping experiment using F_2_ hybrids will map a QTL into a 20- to 40-cM interval [105]. In the RC strains 54% of their donor strain genome reside in segments of medium length (5–25 cM) [106]. However, RC strains can carry on some chromosomes very short segments of the donor strain origin. This feature of the RCS system allowed us previously to narrow the location of *Lmr9* (*Leishmania major* response 9) on chromosome 4 to a segment of 1.9 cM (6.79 Mb) without any additional crosses [101]. The short length of this segment, which controls levels of serum IgE in *L. major* infected mice, enabled us to detect a human homolog of this locus on human chromosome 8q12 and show that it controls susceptibility to atopy [107]. In another study, we were able to precisely map *Tbbr2* (*Trypanosoma brucei brucei* response 2) to 2.15 Mb [53].

In the present F_2_ mapping experiment the shortest locus *Ltr1* is 4.07 Mb long (Figure 2). Although most *Ltr* loci contain several possible candidate genes, here we list (Figure 2)[10], [12], [55]–[83] only those that have been shown previously to influence infection with *Leishmania* ssp.. However, the effects of many of *Ltr* loci might be caused by genes that are at the present not considered as candidates. Currently we are producing mice with recombinant haplotypes that carry individual *Ltr* loci in a very short segment on chromosome. The testing of these strains will restrict the present number of the candidate genes to the most likely ones.

### Conclusion

We present the first description of genetic architecture of response to *L. tropica* in any species. We observed organ specific control of infection and distinct control of parasite load and organ pathology, the typical characteristics of immune response to many pathogens observed in all infections where multiple disease parameters were studied (*L. major* [4], *L. donovani* [4], *Borrelia burgdorferi* [102], *Toxoplasma gondii* [108], *Trypanosoma congolense* [109], and *Chlamydia psittaci* [110]). In addition, the genetic control of response to *L. tropica* exhibits heterogeneity of gene effects, gene-gene interactions, and trans-generational parental effects. These complexities of genetic control have been invoked [111] to explain the very large fraction of heritability that has not been detectable in genome-wide association studies (GWAS) [112], a power deficiency that likely cannot be ameliorated by further increases of the number of tested SNPs or by whole genome sequencing. Identification of these complexities in the present study will open way to elucidation of their functional basis and detection of homologous processes in humans.

## Supporting Information

Table S1 **Numbers of mice analyzed in individual phenotypes.**
(XLS)

Table S2 **Chromosomal positions of typed markers.**
(XLS)

Table S3 **ID numbers of potential candidate genes localized in** ***Ltr*** **loci.**
(XLS)
